# How can UK public health initiatives support each other to improve the maintenance of physical activity? Evidence from a cross-sectional survey of runners who move from *Couch-to-5k* to *parkrun*

**DOI:** 10.1093/heapro/daad108

**Published:** 2023-10-04

**Authors:** Nicola Relph, Michael Owen, Mohammed Moinuddin, Rob Noonan, Paola Dey, Alice Bullas, Helen Quirk, Steve Haake

**Affiliations:** Faculty of Health, Social Care and Medicine, Edge Hill University, St Helens Road, Ormskirk, Lancashire L39 4QP, UK; Faculty of Health, Social Care and Medicine, Edge Hill University, St Helens Road, Ormskirk, Lancashire L39 4QP, UK; Faculty of Health, Social Care and Medicine, Edge Hill University, St Helens Road, Ormskirk, Lancashire L39 4QP, UK; Faculty of Health and Wellbeing, University of Bolton, Deane Road, Bolton, Lancashire BL3 5AB, UK; Faculty of Health, Social Care and Medicine, Edge Hill University, St Helens Road, Ormskirk, Lancashire L39 4QP, UK; The Advanced Wellbeing Research Centre, Sheffield Hallam University, Olympic Legacy Park, 2 Old Hall Road, Sheffield S9 3TU, UK; School of Health and Related Research, The University of Sheffield, 30 Regent Street, Sheffield S1 4DA, UK; The Advanced Wellbeing Research Centre, Sheffield Hallam University, Olympic Legacy Park, 2 Old Hall Road, Sheffield S9 3TU, UK

**Keywords:** physical activity, running, participation, *parkrun*, *Couch-to-5k*

## Abstract

Physical activity improves physical and mental well-being and reduces mortality risk. However, only a quarter of adults globally meet recommended physical activity levels for health. Two common initiatives in the UK are *Couch-to-5k* (an app-assisted 9-week walk/run programme) and *parkrun* (a free, weekly, timed 5-km walk/run). It is not known how these initiatives are linked, how *Couch-to-5k parkrunners* compare to *parkrunners*, and the extent to which this influences their *parkrun* performance. The aims were to compare the characteristics and motives and to compare physical activity levels, *parkrun* performance and the impact of *parkrun* between *Couch-to-5k parkrunners* and *parkrunners*. Three thousand two hundred and ninety six *Couch-to-5k parkrunners* were compared to 55,923 *parkrunners* to explore age, sex, ethnicity, employment status, neighbourhood deprivation, motives, physical activity levels, *parkrun performance* and the impact of *parkrun*. *Couch-to-5k parkrunners* were slightly older, more likely to be female and work part-time, but similar in ethnicity, and neighbourhood deprivation compared with other *parkrunners. Couch-to-5k parkrunners* had different motives for participation and reported high levels of physical activity at registration, which remained to the point of survey completion. This group had slower *parkrun* times but, when registered for a year, completed a similar number of runs (11) per year. Larger proportions of *Couch-to-5k parkrunners* perceived positive impacts compared with other *parkrunners* and 65% of *Couch-to-5k parkrunners* reported improvements to their lifestyle. *parkrun* appears to be an effective pathway for those on the *Couch-to-5k* programme, and the promising positive association between the two initiatives may be effective in assisting previously inactive participants to take part in weekly physical activity.

**CONTRIBUTION TO HEALTH PROMOTION**

- Two popular public health initiatives are *Couch-to-5k* (9 weeks of progressive running) and *parkrun* (a free, weekly, 5-km walk/run in local communities).
- Runners who state *Couch-to-5k* as a reason for *parkrun* participation complete the same number of runs as other *parkrunners.*
- *parkrun* is an effective pathway for those on the *Couch-to-5k* programme, and the positive association may increase the number of older females who take part in weekly physical activity.
- Time-limited physical activity programmes should establish a link to regular community-based activities and may have the potential to attract groups who are typically less active in community-based activities.

## INTRODUCTION

The importance of physical activity for health is well recognized in both academic literature and approaches to public health. There is strong evidence that supports the positive dose–response association between physical activity and physical and mental well-being and mortality for adults (O’Donovan *et al*., 2010; Warburton *et al*., 2010; White *et al*., 2017; Ekelund *et al*., 2019; Strain *et al*., 2020) and children (Lynch, 2019). To achieve good health, the World Health Organization recommends that adults aged 18–64 years should participate in at least 150 min of moderate-intensity aerobic physical activity or at least 75 min of vigorous-intensity aerobic physical activity, or an equivalent combination of moderate- and vigorous-intensity throughout the week (World Health Organization, 2020) Yet only one in four adults globally meet the recommended guidelines (Guthold *et al*., 2018).

The World Health Organization’s Global Action Plan on Physical Activity 2018–2030 (World Health Organisation, 2018) identifies the importance of community-based initiatives to support physical activity participation. In the UK, the National Health Service (NHS) promote a running/walking initiative targeting physically inactive individuals called the *Couch-to-5k*. The aim of this nine-week progressive programme is to increase physical activity levels using a free downloadable mobile ‘app’ that people can use at a time that suits them (NHS, 2020). The programme involves three runs/walks per week, with one day of rest in between, which varies from week to week (NHS, 2020). The creator, Josh Clark, wanted to create a bridge between walking and running, gradually progressing to the final week, a 30-min continuous run (NHS, 2020). But for some, this may not result in achieving a 5-km run as alluded to in the name of the initiative. Therefore, people may look to an alternative running programme that is not time limited to continue their running participation.

*parkrun* is a weekly, free to enter, 5-km mass participation event delivered across 22 countries (parkrun.com) that has been taking place in the UK since 2004. The event has a strong ethos of inclusivity, social interaction and community (Hindley, 2018) and is used by General Practices in the UK and Ireland as a Public Health referral option (Fleming *et al*., 2020). *parkrun* can be completed by running, walking or a combination of both and attracts people of all ages and abilities including those with limited experience of running (Stevinson and Hickson, 2014; Haake, 2018; Quirk and Haake, 2019). Indeed, the average parkrun time is approximately 29 min, thus roughly half complete the 5km slower than 30 min and may attract *Couch-to-5k* participants. Furthermore, *parkruns* are delivered by local teams of volunteers and participants are also encouraged to volunteer to complete roles such as marshalling, timekeeping, scanning barcodes, handing out finish tokens or tail walking; these volunteers are not required to ever run/walk the event.

Mass community-based participation events including *parkrun* have been shown to increase physical activity levels (Heath *et al*., 2012; Cleland *et al*., 2019), and cardiorespiratory fitness levels over 12 months (Stevinson and Hickson, 2019). Furthermore, previous research has shown that group support and social interaction, which may be provided at *parkrun* (Grunseit *et al*., 2020), are crucial to physical activity adherence following a beginner running/walking programme (Wiltshire and Stevinson, 2018) such as the *Couch-to-5k* programme.

Presently, little is known about the characteristics and physical activity patterns of *Couch-to-5k parkrunners*. It would be useful for research and public health practice to understand how many progress onto *parkrun* and engage in the *parkrun* initiative in the long term. However, the extent to which *Couch-to-5k* serves as a pathway into *parkrun* is currently unknown. Furthermore, evidence on physical activity in general has identified that women, people living in deprived areas and older people with chronic diseases are more likely to be inactive (World Health Organisation, 2018). There is one exploratory study to date that found *parkrun* does attract these underrepresented groups (Stevinson and Hickson, 2014). However, other research identified that *parkrunners* are still more likely to be white, have higher socio-economic status and already be active (Fullagar *et al*., 2020; Grunseit *et al*., 2020). Therefore, another important finding from this study will be to see whether there is potential for the *Couch-to-5k* initiative to not only offer a pathway into *parkrun* but to also increase the diversity of this mass participatory event and improve physical activity levels of marginalized groups with typically lower physical activity levels. Therefore, the aims of this study are as follows:

1.) To compare the socio-demographic characteristics and participation motives between a sub-group of *Couch-to-5k parkrunners* and *parkrunners*.
2.) To compare the physical activity levels, *parkrun* performance measures and the impact of *parkrun* between *Couch-to-5k parkrunners* and *parkrunners*.

## METHODS

### Research design, procedures and participants

The current study was comparative cross-sectional. The *parkrun* Health and Wellbeing Survey 2018: UK (Haake *et al*., 2018) was distributed online to all registered *parkrunners* in the UK aged 16 or over (2.2 million) between October and December 2018. The survey included a maximum of 47 questions, all were optional apart from identification of role in parkrun; either runners/walkers, runners/walkers who also volunteered at *parkrun* or volunteers only, one current health condition, disability or illness question and two life satisfaction questions. Full details of survey development and data handling processes are reported elsewhere (Quirk *et al.*, 2021).

A total of 100,864 individuals initially responded to this survey (4.5% participation rate), however once incomplete responses and volunteers only were removed the sample size was 59,999 *parkrunners*. For the current study, *parkrunners* who reported ‘it was part of a *Couch-to-5k programme*’ as one of their top three motives for participation in *parkrun* as a runner/walker were included in a sub-group analysis (herein referred to as ‘*Couch-to-5k parkrunners*’). This sub-group comprised 3,296 people (5.5% of those who responded to the original survey).

### Outcome measures

Supplementary File 1 details all outcome measures, the full survey and a copy of the participant information sheet provided to all participants. The outcome measures used in the current study are detailed below.

#### Socio-demographic characteristics

Participants reported their date of birth and hence age, sex, ethnicity and employment status. Neighbourhood deprivation was calculated from participant-reported postcodes provided at *parkrun* registration using English Indices of Multiple Deprivation (IMD) for lower-layer super output areas (Ministry of Housing, 2019). These scores were then collapsed into quartiles ranging from the most (level 1) to the least (level 4) deprived.

#### Motives

Participants were asked *What motivated you to first participate at parkrun as a runner or walker?* Respondents were asked to select a maximum of three answers out of a possible 21 motives. The answer choices were displayed in randomized order to help reduce response bias. The final choice was ‘other’, and respondents were asked to specify their motive. Participants were placed in the *Couch-to-5k* sub-group if they selected ‘it was part of a *Couch-to-5k programme*’ as one of their three motives.

#### Physical activity levels

Self-reported physical activity level at *parkrun* registration was collected using the following question: *Over the last 4 weeks*, *how often have you done at least 30 min of moderate exercise (enough to raise your breathing rate)?* Response options were as follows: (i) less than once per week; (ii) about once per week; (iii) about twice per week; (iv) about three times per week; (v) four or more times per week; and (vi) rather not say/do not know. Participants were asked this question again at the time of the survey to calculate the change in physical activity *since* registration.

#### *parkrun* performance

Participants provided their *parkrun* ID number (a unique ID number provided to all *parkrun* registrants to identify them on the *parkrun* database and enable the collation of all their *parkrun* participation data). This ID (or their name, DOB, home parkrun—if their ID was not provided) was then matched to their *parkrun* profile and provided mean *parkrun* time, the number of years registered, total number of *parkruns* completed since registration and *parkruns* completed per year (if registered more than 1 year).

### Data analysis

Frequency and percentage were used as descriptive statistics for categorical variables and median with interquartile range were used to summarize the continuous variables. Median was chosen because variables were highly skewed. Comparisons between *Couch-to-5k parkrunners* and the remaining *parkrunners* were analysed using Mann–Whitney *U* and Pearson’s chi-squared tests with accompanying effect sizes. Significance was accepted at *p* < 0.05 level. If *p* was calculated as <0.001, we have reported this as such.

## RESULTS

### Socio-demographic characteristics

*Couch-to-5k parkrunners* were older than other *parkrunners* (median 50.5 years compared with 48.8 years; *p* < 0.001, effect size = 0.03) and more likely to be female than other *parkrunners* (72.5% vs 50.5%, *p* < 0.001, effect size = 0.10). Other *parkrunners* were predominantly white with 3.0% from BAME backgrounds and *Couch-to-5k parkrunners* were not significantly different to this (*p* > 0.05). However, *Couch-to-5k parkrunners* were more likely to be in part-time employment (19.7% vs 13.4%) and less likely to be in full-time paid employment (51.5% vs 54.8%) or self-employed (7.4% vs 9.4%) compared with other *parkrunners* (*p* < 0.001, effect size = 0.05); the proportion who were retired were similar for both (12.0% vs 12.2%, *p* > 0.05). A total of 9.5% of the other *parkrunners* came from the most deprived neighbourhoods and 40.1% from the least deprived: there was no difference between *Couch-to-5k parkrunners* or other *parkrunners* (*p* > 0.05).

### Motives for *parkrun*

Overall, 5.5% of total survey respondents chose *Couch-to-5k* as a motive. The top motives were fitness, physical health and sense of personal achievement (see Table 1). Motives for *Couch-to-5k parkrunners* tended to be ranked in the same order as other *parkrunners* but with lower proportions; this is probably due to the limit of three motives per person (i.e. only two additional motives to choose for *Couch-to-5k parkrunners*). Despite this, *Couch-to-5k parkrunners* may have been less motivated to first participate because of fitness (35.3% vs 57.4%; χ^2^ = 627.1, *p* < 0.001, effect size = 0.10) to feel part of a community (3.9% vs 11.4%, *Χ*^*2*^ = 182.5, *p* < 0.001, effect size = 0.06) and to spend time outdoors (3.1% vs 10.8%; *Χ*^*2*^ = 201.9, *p* < 0.001, effect size = 0.06). Ranked third as a motive, *Couch-to-5k parkrunners* were equally likely to select a sense of personal achievement when compared with the rest of the sample (27.7% vs 26.8%; *p* = 0.270).

**Table 1:** Comparison of motives for first participating in *parkrun* using Pearson’s chi-squared tests. *n =* number of participants, **χ**^2^ = chi-squared statistic, *p = p*-value, ϕ = effect size

Motive	*Couch-to-5k*	Rest of sample	Total	χ^2^	*p*	ϕ
*n*	3296	55 923	59 261			
*Couch-to-5k*	100%	0%	5.4%			
Fitness	35.3%	57.4%	56.2%	627.1	<0.001	0.10
Physical health	31.1%	35.1%	35.0%	3.4	0.067	0.01
Sense of personal achievement	27.7%	26.8%	26.9%	1.2	0.270	0.00
My friends and colleagues wanted me to	11.9%	15.3%	15.2%	28.7	<0.001	0.02
Mental health	10.3%	13.1%	13.0%	22.5	<0.001	0.02
To feel part of a community	3.9%	11.4%	11.0%	182.5	<0.001	0.06
To manage a health condition	3.6%	3.4%	3.4%	0.4	0.535	0.00
To spend time outdoors	3.1%	10.8%	10.3%	201.9	<0.001	0.06
To spend time with family	2.9%	7.5%	7.3%	100.2	<0.001	0.04
Happiness	2.9%	6.9%	6.7%	81.1	<0.001	0.04
To spend time with friends	2.4%	8.1%	7.7%	141.7	<0.001	0.05
To meet new people	2.0%	4.3%	4.1%	41.8	<0.001	0.03

### Physical activity levels

Physical activity at registration for *Couch-to-5k parkrunners* was significantly different to other *parkrunners* (*p* < 0.001, effect size = 0.10). Those who did up to 1 day of activity per week at registration represented 10.8% of the *Couch-to-5k parkrunners* compared with 16.9% for other *parkrunners*; additionally, 52.1% of *Couch-to-5k parkrunners* did about 3 days of activity at registration, compared with 32.6% other *parkrunners*.

Table 2 shows the change in physical activity from registration to the point of the survey. Just over a third (33.7%) of *Couch-to-5k parkrunners* increased their activity category level, while 22.5% decreased it; 43.8% stayed the same. Thus, there were 1.5 times as many *Couch-to-5k parkrunners* who increased their activity as decreased it. In comparison, 41.7% of the other *parkrunners* increased their activity while 16.5% decreased it, a ratio of 2.5. The distribution for the *Couch-to-5k parkrunners* and the rest of the sample was different at *p* < 0.001 (see Table 2 for corresponding effect sizes).

**Table 2:** Comparison in change in physical activity in categories using Pearson’s chi-squared tests. χ^2^ = chi-squared statistic, *p* = *p*-value, ϕ = effect size

Change in category	*Couch-to-5k*	Rest of sample	Total	*Couch-to-5k*	Rest of sample	Total	Change in category	*Couch-to-5k*	Rest of sample	Total
−4	7	98	105	0.3%	0.2%	0.2%				
−3	41	429	470	1.6%	1.1%	1.1%				
−2	123	1285	1408	4.7%	3.1%	3.2%				
−1	421	4920	5341	16.0%	12.0%	12.3%	Decreased	22.5%	16.5%	16.8%
Stayed the same	1150	17 071	18 221	43.8%	41.8%	41.9%	Stayed the same	43.8%	41.8%	41.9%
1	617	10 950	11 567	23.5%	26.8%	26.6%	Increased	33.7%	41.7%	41.2%
2	191	4391	4582	7.3%	10.7%	10.5%				
3	61	1350	1411	2.3%	3.3%	3.2%	χ^2^	105.4		
4	17	353	370	0.6%	0.9%	0.9%	*p*	<0.001		
Total	2628	40 847	43 475	100%	100%	100%	ϕ	0.05		

### *parkrun* performance

**Fig. 1:**
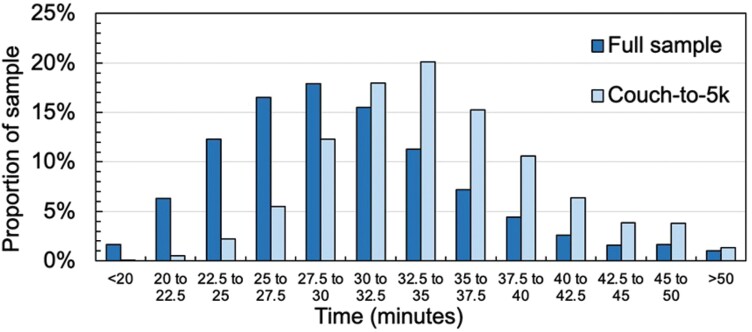
Comparison of average time to complete *parkrun* between *Couch-to-5k parkrunners* (labelled as Couch-to-5k) and other *parkrunners* (labelled as Full sample).

The mean completion time of *Couch-to-5k parkrunners* was significantly longer at 34 min 36 s (SD = 5.7 min) compared with 29 min 52 s (SD = 6.2 min) for the rest of the sample (Figure 1: *F* = 15.4, *p* < 0.001, effect size *d* = 0.74). *Couch-to-5k parkrunners* were registered for a median of 1.21 years compared with 2.74 years for the rest of the sample (*p* < 0.001, effect size = 0.13) and hence had completed less *parkruns* (a median of 11 vs 22; *p* < 0.001, effect size = 0.10). However, when only considering participants who had been registered for at least a year (*Couch-to5k parkrunners*, *n* = 1429, rest of the sample, *n* = 32 782), the *Couch-to-5k parkrunners* had completed the same number of *parkruns* per year as the rest of the sample at approximately 11 per year (*p* > 0.05).

### The impact of *parkrun*

The impact of running/walking for *Couch-to-5k parkrunners* compared with the rest of the sample is shown in Table 3, showing the proportions who indicated *better* and *much better* for each measure. All measures were significantly different at *p* < 0.001 between *Couch-to-5k parkrunners* and the remaining sample.

**Table 3:** Impact of *parkrun*. Comparison of those reporting *better and much better* using Pearson’s chi-squared tests. χ^2^ = chi-squared statistic, *p* = *p*-value, ϕ = effect size

	*Couch-to-5k*	Rest of sample	Total	*Couch-to-5k*	Rest of sample	Total	χ^2^	*p*	ϕ
Sense of personal achievement	3153	53 122	56 275	96.4%	90.4%	90.7%	628.3	<0.001	0.11
Fitness	3146	53 122	56 268	95.6%	89.0%	89.3%	567.5	<0.001	0.10
Physical health	3148	53 113	56 261	92.5%	84.2%	84.7%	425.8	<0.001	0.09
Happiness	3151	53 065	56 216	83.4%	78.5%	78.7%	84.0	<0.001	0.04
Enjoyment of the outdoors	3151	53 099	56 250	82.9%	73.6%	74.1%	408.8	<0.001	0.09
Mental health	3144	53 070	56 214	74.7%	68.9%	69.2%	117.2	<0.001	0.05
Confidence	3150	53 074	56 224	73.0%	60.6%	61.3%	260.9	<0.001	0.07
Being active in a safe environment	3151	53 041	56 192	71.7%	59.3%	59.9%	281.5	<0.001	0.07
Your enjoyment of competing	3146	53 106	56 252	69.9%	72.9%	72.6%	51.7	<0.001	0.03
Feeling part of a community	3147	53 069	56 216	69.7%	69.6%	69.7%	26.1	<0.001	0.02
Lifestyle	3145	53 063	56 208	65.0%	51.0%	51.7%	253.5	<0.001	0.07
Ability to manage my weight	3143	53 064	56 207	64.7%	51.6%	52.3%	283.4	<0.001	0.07
Number of new people you meet	3154	53 082	56 236	63.5%	57.2%	57.5%	78.7	<0.001	0.04
Time spent with friends	3146	53 034	56 180	42.1%	41.1%	41.1%	14.5	0.006	0.02
Time spent with family	3147	52 992	56 139	26.4%	27.9%	27.7%	13.6	0.008	0.02

The three largest proportions perceiving improvement were for *a sense of personal achievement* (96.4% vs 90.4%, diff = 6.0%), *fitness* (95.6% vs 89.0%, diff = 6.6%) and *physical health* (92.5% vs 84.2%, diff = 8.3%). All measures had a larger proportion reporting better and much better for *Couch-to-5k parkrunners*, except *your enjoyment of competing* (69.9% vs 72.9%, diff = −3.0%).

The other largest differences were for *the enjoyment of the outdoors* (82.9% vs 73.6%, diff = 9.3%; χ^*2*^ = 408.8, effect size = 0.09), *being active in a safe environment* (71.7% vs 59.3%, diff = 12.4%; χ^2^ = 408.8, effect size = 0.07), *confidence* (73.0% vs 60.6%, diff = 12.4%; χ^2^ = 260.9, effect size = 0.07), *ability to manage my weight* (64.7% vs 51.6%, diff = 13.1%; χ^2^ = 283.4, effect size = 0.07) and *lifestyle* (65.0% vs 51.0%, diff = 14.0%; χ^2^ = 253.5, effect size = 0.07).

## DISCUSSION

The aims of the study were to compare the socio-demographic characteristics and participation motives between a sub-group of *Couch-to-5k parkrunners* and *parkrunners* and to compare the physical activity levels, *parkrun* performance measures and the impact of *parkrun* between *Couch-to-5k parkrunners* and *parkrunners.* We did this in order to establish whether *parkrun* provides an effective pathway for people who have previously been inactive to continue physical activity following the *Couch-to-5k* programme.

This study identified that *Couch-to-5k parkrunners* were slightly older, more likely to be female and be in part-time employment, but with similar ethnicity (mainly white) and neighbour deprivation levels compared with other *parkrunners*. Ages in the current study are similar to previous *parkrun* research, between 35 and 54 years (Cleland *et al*., 2019; Stevinson and Hickson, 2019; Fullagar *et al*., 2020). Hence, it appears both initiatives are likely to attract middle- to older-aged groups, which is important as it is known that these groups have higher levels of inactivity (Pinto Pereira *et al*., 2018).

*Couch-to-5k parkrunners* were more likely to be female compared with other *parkrunners*. Similarly, a recent study on the *Couch-to-5k* programme reported more female participants than male participants (Relph *et al*., 2020). Previous research in the UK highlighted that *parkrun* may be more likely to attract participation from males (Stevinson and Hickson, 2014). Thus, the route from the *Couch-to-5k* programme into *parkrun* may be an effective avenue to increase physical activity among females. Stride *et al*. (2020) suggest inclusivity may help females participate in *parkrun,* for example the flexibility, low time commitment and family atmosphere of *parkrun* events. The perception that the tail walker volunteer role (the last person to cross the finish line, ensuring that everyone is accounted for) is always female may also help women feel more welcome and confident to participate in *parkrun* (Stride *et al*., 2020). This is a positive finding that suggests a potentially important association between the two running interventions to attract more females to sustain physical activity after completion of the time-limited *Couch-to-5k* programme.

Employment levels were similar in the current study, which corresponds to other research, with most participants across the whole sample employed (Cleland *et al*., 2019; Fullagar *et al*., 2020). White British was the main ethnicity in this study and other UK *parkrun* samples (Grunseit *et al*., 2020), and participants were mainly from neighbourhoods with low levels of deprivation, although areas of higher deprivation were represented. Smith *et al*. (2020) reported that areas in England of higher ethnic diversity and IMD have lower levels of *parkrun* participation even when controlling for population density, distance to the nearest *parkrun* event and age of population. The *parkrun* organizations are aware of this lack of diversity and keen to address this through initiatives such as their Outreach Ambassador Programme (Fullagar *et al*., 2020).

*Couch-to-5k parkrunners* were less motivated by fitness, feeling part of a community, spending time outdoors, spending time with family or spending time with friends compared with other *parkrunners*. This may be because of their prior training and community created by the *Couch-to-5k* programme. However, future work could explore these findings using more qualitative methods.

The majority of *Couch-to-5k parkrunners* did approximately 3 days per week of activity at registration; this was likely because of the *Couch-to-5k* programme, which has participants doing this frequency of activity by the end of the nine weeks. As a consequence, *Couch-to-5k parkrunners* were less likely to increase activity after *parkrun* participation. This is a positive finding for the *Couch-to-5k* programme. The other *parkrunners* also reported good levels of physical activity levels, with more participants increasing activity levels in the time from registration to survey completion than *Couch-to-5k parkrunners*. Research supports this finding; UK *parkrunners* self-reported on average 350 min per week of moderate- to vigorous-intensity activity with only 8.8% reporting below recommended physical activity thresholds for health maintenance (Stevinson and Hickson, 2019). Stevinson and Hickson (2014) reported that most *parkrunners* in the UK classified themselves as regular runners (48%). Hence, the association between the two initiatives appears to help maintain regular physical activity levels.

The current study suggests *Couch-to-5k parkrunners* may not be considered inactive participants, a global target public health population (World Health Organisation, 2018). This may be explained in part as the *Couch-to-5k* programme involves running three times a week prior to participation in *parkrun*. Fullagar *et al*. (2020) note that perceptions of the ‘run’ in the name ‘*parkrun’* may be a barrier to inactive participants who may be unaware that it is possible to walk the full 5-km route at each event. However, *parkrun* does attract smaller proportions of inactive people (Quirk *et al.*, 2021). Future research should consider how less active people could be attracted to take part in both physical activity initiatives.

*Couch-to-5k parkrunners* on average took longer to complete the *parkrun* but did the same number of *parkruns* per year (at just less than one per month) when compared with other *parkrunners*. Therefore, it appears that *Couch-to-5k parkrunners* are similarly integrated into *parkrun* as other *parkrunners*. This is an important finding as it demonstrates that one, time-restricted physical activity intervention, can be successfully linked to another physical activity intervention to maintain activity levels.

Almost all impact measures of *parkrun* relating to health and well-being were greater for *Couch-to-5k parkrunners* compared with the rest of the sample, with sense of personal achievement, fitness and physical health being the top three improvements. The *Couch-to-5k* programme is designed to attract those who are inactive, and hence may explain why almost all the sub-group deemed these to improve. Furthermore, the sub-group did not view competition as an important impact, again, likely due to the less performance-orientated nature of this group.

### Limitations

The findings should be considered in light of the following methodological limitations. The study is a cross-sectional design, which limits the ability to report cause and effect and there may have been a selection bias effect as recruitment was not random. Furthermore, the survey relies on some self-reported measures, which can introduce recall error and response bias. However, it is important to note that the outcome measure of number of *parkruns* completed was measured using *parkrun* ID, which is recorded at each event. Finally, the sub-group of *Couch-to-5k parkrunners* was generated based on participants listing the *Couch-to-5k* as one of their top three motives for doing *parkrun.* Therefore, this sub-group may have missed *parkrunners* who came from the *Couch-to-5k* but did not list it in their top three motives.

## CONCLUSION

Around 5% of our *parkrun* sample identified *Couch-to-5k* as a motive for first participating as a runner or walker at parkrun. This group appears to be largely female, around 50 years of age and similar in ethnicity, employment status and neighbourhood deprivation levels to other *parkrunners*. The majority registered with around 3 days of activity per week similar to the *Couch-to-5k* programme. *Couch-to-5k parkrunners* appear to go on to become integrated *parkrunners*, doing the same number of parkruns per year as others (a median of 11 per year). They were less motivated by fitness and improving social connections, but almost all impact measures relating to health and well-being were greater for *Couch-to-5k parkrunners* including fitness, physical health, mental health, ability to manage their weight and lifestyle. *parkrun* appears to be an effective pathway for those on the *Couch-to-5k* programme and the association between the two running initiatives may be effective in increasing the number of females who take part in weekly physical activity.

The findings of this study have important implications for future public health initiatives that aim to increase and sustain physical activity levels. First, programmes that are time limited, such as *Couch-to-5k*, should establish a link with a local *parkrun* to provide regular opportunities for participation in activity and continued opportunities to experience the multiple health benefits of a mass participatory event. If a *parkrun* is not available, regular community-based activity should be embedded at the end of a short-term programme. Second, beginner, time-limited programmes like *Couch-to-5k* have the potential to attract groups who are typically less active, such as women, to community-based events like *parkrun* which provide opportunities to sustain levels of physical activity.

## Supplementary Material

daad108_suppl_Supplementary_Material
